# Author Correction: The genetic and environmental composition of socioeconomic status in Norway

**DOI:** 10.1038/s41467-025-60803-4

**Published:** 2025-06-19

**Authors:** Joakim Coleman Ebeltoft, Espen Moen Eilertsen, Rosa Cheesman, Ziada Ayorech, Arno Van Hootegem, Torkild Hovde Lyngstad, Eivind Ystrom

**Affiliations:** 1 PROMENTA Center, Department of Psychology, University of Oslo, Oslo, Norway; 2 Department of Sociology & Human Geography, University of Oslo, Oslo, Norway; 3 Centre for Fertility and Health, Norwegian Institute for Public Health, Oslo, Norway; 4 PsychGen Centre for Genetic Epidemiology and Mental Health, Norwegian Institute of Public Health, Oslo, Norway; 5 Centre for Research on Equality in Education, Faculty of Education, University of Oslo, Oslo, Norway

**Keywords:** Society, Genetic association study, Sociology, Education, Behavioural genetics

Correction to: *Nature Communications* 10.1038/s41467-025-58961-6, published online 14 May 2025

In the version of the article initially published, in the Results “Extracting shared environment contributions” section, there were mistakes in figure citations. The first three citations to Fig. 3 in the current version originally cited Fig. 2, while the citation to Fig. 4 originally cited Fig. 3. The errors are corrected in the HTML and PDF versions of the article.

