# Supplementary material for: The genetic and environmental composition of socioeconomic status in Norway
Source: Nat Commun. 2025 May 14;16:4461. doi: 10.1038/s41467-025-58961-6 (PMC12078464; doi:10.1038/s41467-025-58961-6)

Supplementary Information

Descriptive information about the Norwegian Mother, Father, and Child Cohort

The sample is drawn from the Norwegian Mother, Father, and Child Cohort Study (Moba), which recruited pregnant mothers, corresponding children and fathers from across Norway between 1999 and 2008. Although the participants are from all corners of the country, the sample is not fully representative of the population of parents of children born between 1999 and 2008. Educational attainment, occupational prestige, income and wealth are upward biased in the Moba sample compared to the population. Compared to the Norwegian population, Moba is higher educated with a ratio of 1.4 times more people with a bachelor's degree and a ratio of 1.5 times more people with a master's degree. The mean occupational prestige difference between the Moba and population parents is equivalent to the difference between a nurse with and without specialization. Their mean income is 11% higher and their mean gross wealth is 16% higher. The Moba sample also have their first child a year later and 10% more have a cohabiting partner at age 40. The birth year of the Moba parents ranges from 1937 to 1992; the mode is 1974, the mean is 1973, and the standard deviation is 5 years. For in-depth tables and graphs describing the demographic differences between the Norwegian population and Moba sample see the supplementary information.

Supplementary Data S1 (in own excel file): Demographic characteristics of population and samples. The table shows that our sample has a higher proportion of parents with a cohabitant than the population. The age at first birth is slightly older in the sample while the number of children is similar in the sample.

Supplementary Table S2: Heritability estimates. The table shows estimates and S.E. of educational attainment, occupational prestige, income, and wealth corresponding to Figure 2 in main article.

**Method**	**SES Indicator**	**Heritability estimate**	**Standard error**
Family Pedigree AE	Educational attainment	72.99%	0.89%
	Occupational status	40.26%	1.97%
	Income	51.41%	2.31%
	Wealth	57.63%	0.89%
Family Pedigree ACE<br>Standard C assumptions	Educational attainment	72.61%	1.50%
Sibling r_c = 1	Occupational status	35.30%	1.14%
Cousin r_c = 0	Income	45.29%	1.44%
	Wealth	55.57%	1.38%
Family Pedigree ACE			
Sibling r_c = 1	Educational attainment	39.98%	3.42%
Estimated cousin r_c = 0.59	Occupational status	33.97%	1.46%
	Income	29.97%	2.76%
	Wealth	24.87%	2.65%
IBD	Educational attainment	69.24%	1.87%
	Occupational status	36.15%	1.81%
	Income	41.48%	1.79%
	Wealth	52.04%	2.20%
GCTA-GREML	Educational attainment	23.46%	1.06%
	Occupational status	17.80%	1.06%
	Income	6.53%	0.97%
	Wealth	11.98%	1.06%
LD score regression	Educational attainment	19.89%	0.81%
	Occupational status	14.95%	0.76%
	Income	6.78%	0.48%
	Wealth	8.02%	0.59%

Figure S1: Histogram of birth year of sample. Each bin is 5 years.

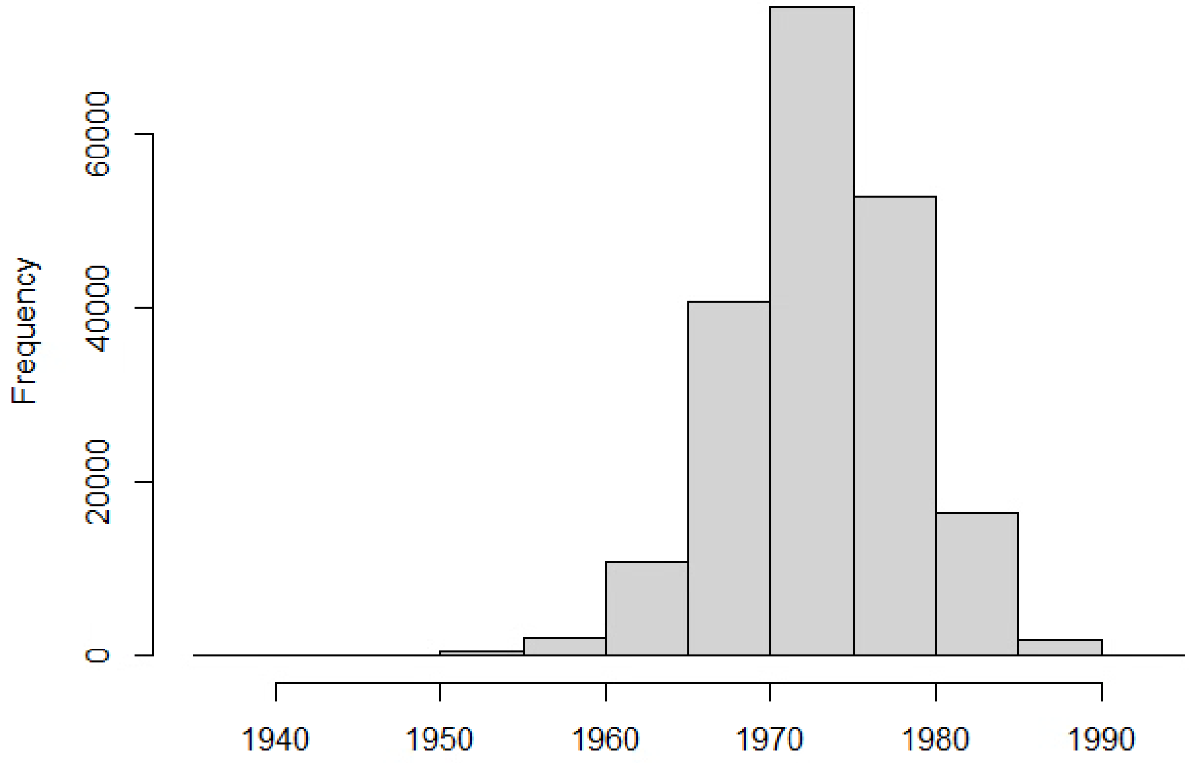

Table S3: Educational attainment in population and sample in both years and ISCED category. Years were used in the analysis. N=number.

	**Population**	**Population**	**Sample**	**Sample**
	**Women**	**Men**	**Women**	**Men**
**N (%) education 0 yrs /ISCED11 cat 0**	0	0	0	0
**N (%) education 7yrs/ ISCED11 cat 1**	2,700 (0.60%)	2,190 (0.51%)	82 (0.086%)	53 (0.071%)
**N (%) education 10 yrs / ISCED11 cat 2**	50880 (11.3%)	62627 (14.5%)	5,734 (6.03%)	6,447 (8.59%)
**N (%) education 13 yrs / ISCED11 cat 3**	116941 (26.0%)	157785 (36.4%)	21798 (22.9%)	26099 (34.8%)
**N (%) education 14.5 yrs / ISCED11 cat 4**	9627 (2.14%)	20812 (4.80%)	2108 (2.22%)	4189 (5.58%)
**N (%) education 16 yrs / ISCED11 cat 5**	151278 (33.6%)	94080 (21.7%)	44942 (47.2%)	22713 (30.3%)
**N (%) education 18 yrs / ISCED11 cat 6**	44162 (9.82%)	45368 (10.5 %)	14446 (15.2%)	11981 (16.0%)
**N (%) education 21 yrs / ISCED11 cat 7**	4734 (1.05%)	6409 (1.48 %)	1356 (1.42%) Ratio 1.35	1510 (2.01 %)

Table S3 shows that the sample is higher educated than the population. The sample has a substantial higher degree of people with a bachelor's (ratio of 1.4) and master's degree (ratio of 1.5), and hardly anyone with only elementary school as their highest formal education.

In the sample, the correlation between highest obtained educational level achieved between ages 35 and 45 and the highest acheved educational level regardless of age was *r*=.95.

Figure S2: Average SIOPS occupational status between ages 35 and 45. Mean = dotted line.

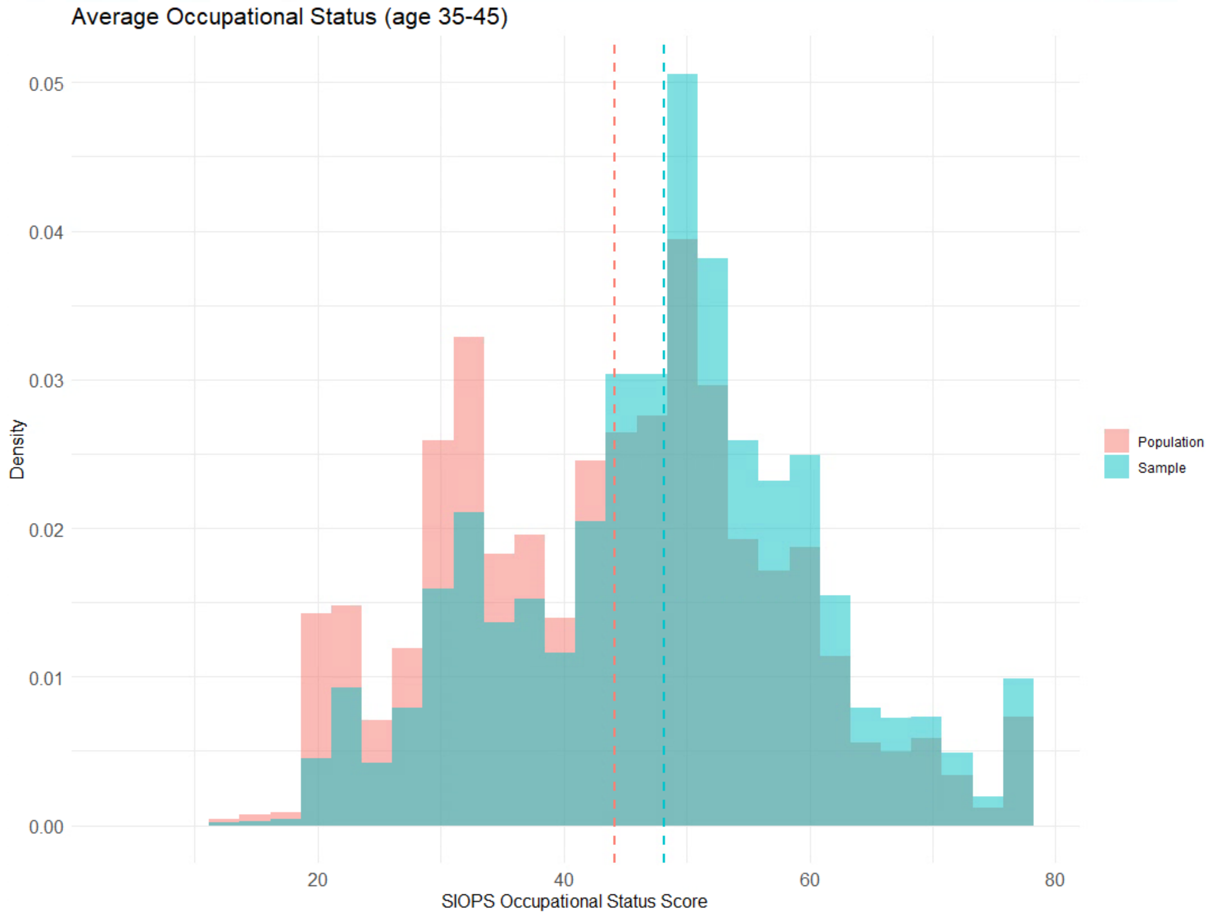

Fig. S2 shows the difference between the population and the sample with regards to the SIOPS occupational status index. The average occupational status between ages 35 and 45 was 44 (SD=13.4) in the population and 48 (SD =12.6) in the sample. This is equivalent to the difference between a nurse (SIOPS score = 44) and a nurse with specialization (SIOPS score = 48). Median was 45 in the population and 50 in the sample.

The intraclass correlation (ICC) for occupational prestige across ages 35 and 45 within a person, adjusted for year, birthyear, and sex, was *r*=.80.

Figure S3: Average total income after tax in sample and population between ages 35 and 45. Income is inflation adjusted in NOK. Total income after tax over 1e6 and under 0 were truncated to fit the boundaries of the graph. Mean = dotted line.

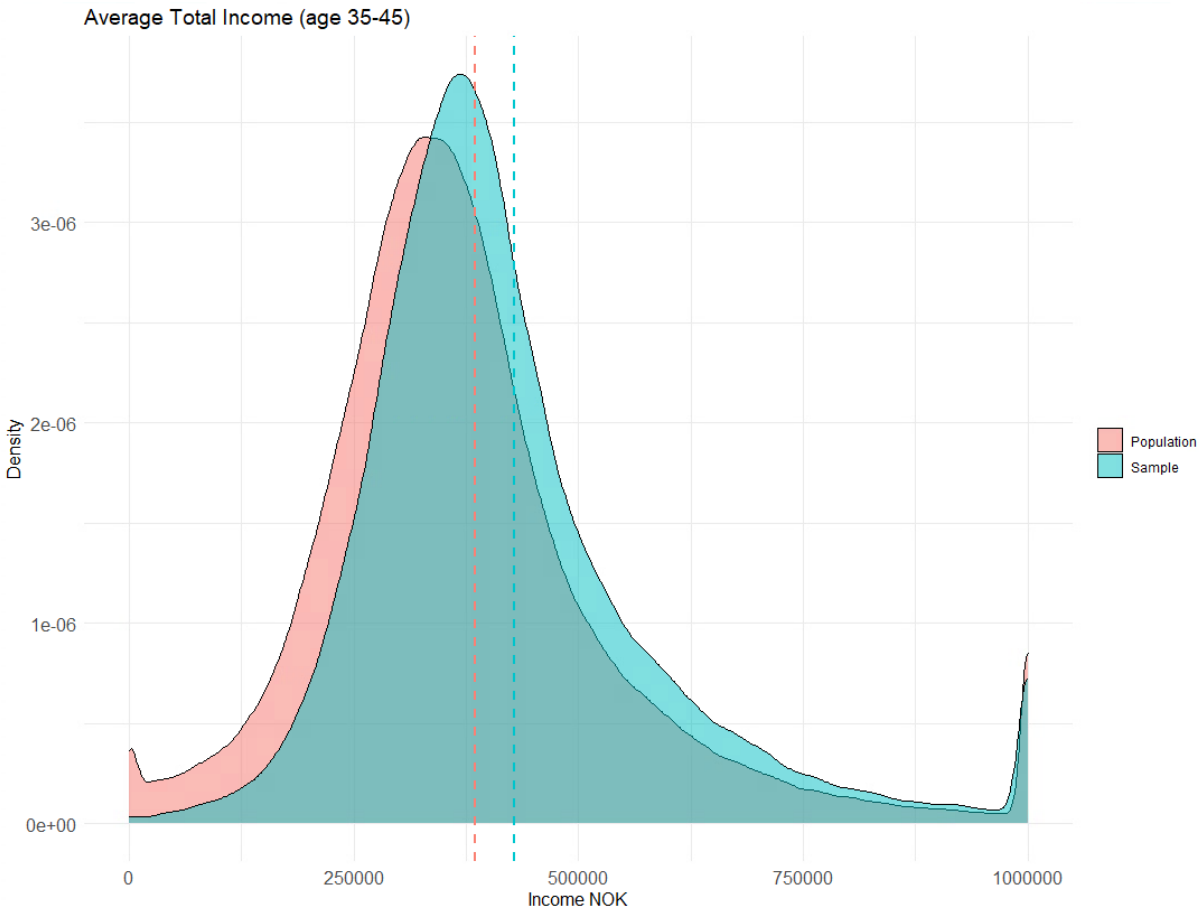

Fig. S3 shows the difference between the population and the sample with regards to total income after taxes. The average total income after taxes between ages 35 and 45 was 384,777 NOK (SD=431,051) in the population and 428,250 NOK (SD=469,942) in the sample, a difference equivalent to an extra month of salary. Median income is 350,518 NOK in the population and 387,529 NOK in the sample.

The intraclass correlation (ICC) for income across ages 35 and 45 within a person, adjusted for year, birthyear, and sex, was *r*=.13.

Figure S4: Average gross wealth between ages 35 and 45. Gross wealth is adjusted for inflation and in NOK. Averages under 0 and over 1e7 were truncated to fit the boundaries of the graph. Mean = dotted line.

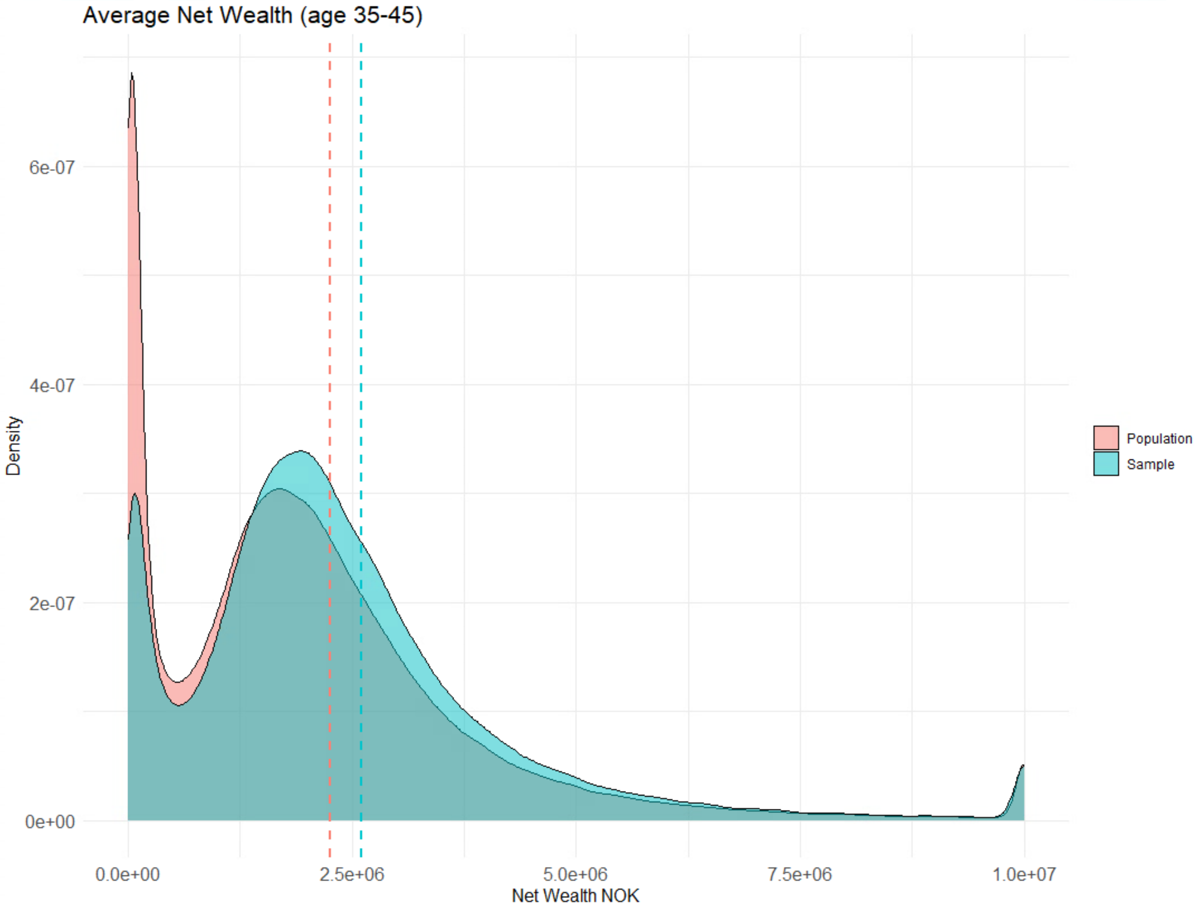

Fig. S4 shows the difference between the population and the sample with regards to gross wealth. The average gross wealth between ages 35 and 45 was 2,249,025 (SD=11,237,860) NOK in the population and 2,599,882 (SD=7,894,753) NOK in the sample, a ratio of 1.16. The median gross wealth is 1,759,679 NOK for the population and 2,076,721 NOK for the sample.

The intraclass correlation (ICC) for wealth across ages 35 and 45 within a person, adjusted for year, birthyear, and sex, was *r*=.76.

Assumptons of first cousin shared environment correlations

Figure S5: A, C, and E contribution to occupational prestige under different shared environment correlation assumptions. Additive gegrossic (h2), share environmental (c2), and non-shared environmental (e2) effects on occupational prestige across assumptions of first cousin shared environment correlation. Sibling shared environment correlation was assumed to be 1. Intervals between assumptions are .05 and ribbons represent 95% confidence intervals.

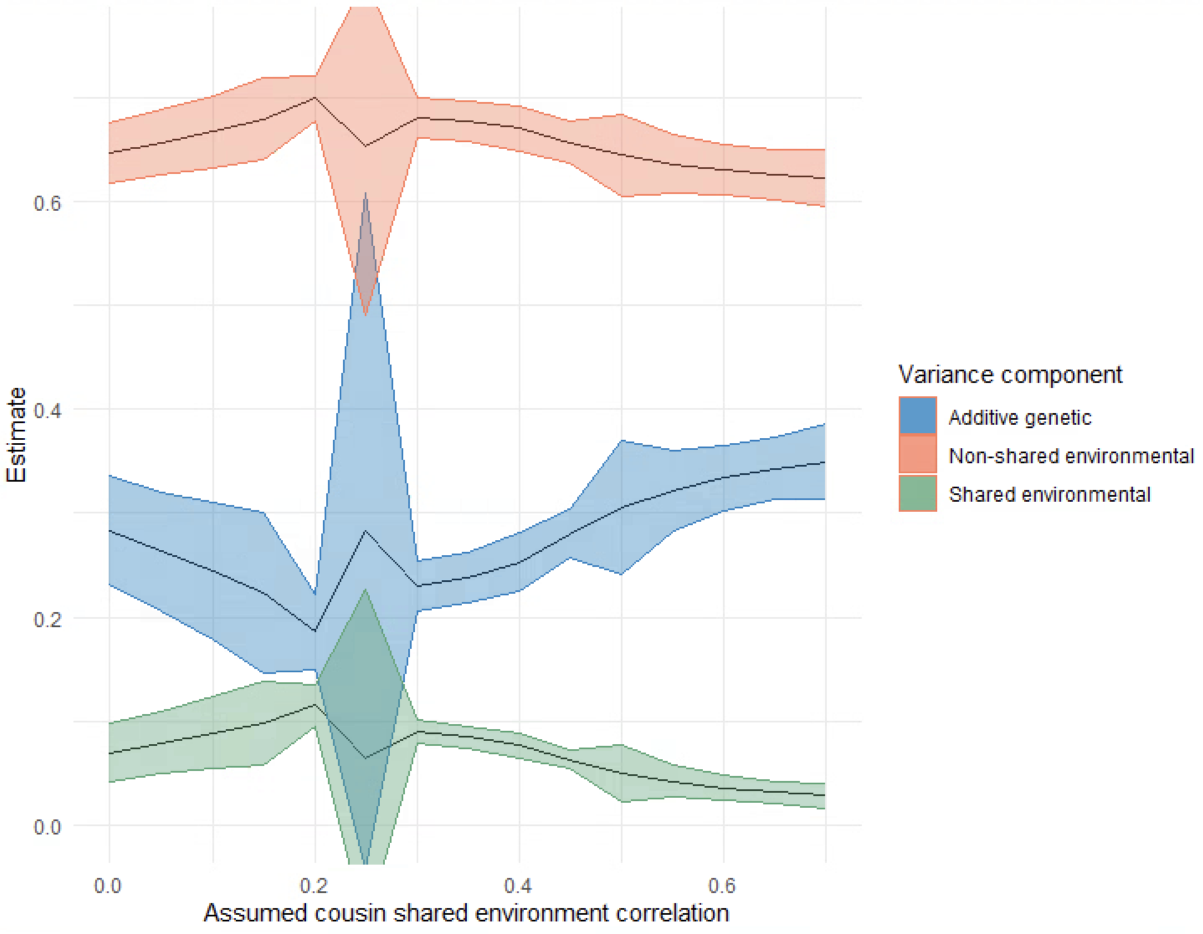

Figure S6: A, C, and E contribution to income under different shared environment correlation assumptions. Additive genetic (h2), share environmental (c2), and non-shared environmental (e2) effects on income across assumptions of first cousin shared environment correlation. Sibling shared environment correlation was assumed to be 1. Intervals between assumptions are .05 and ribbons represent 95% confidence intervals.

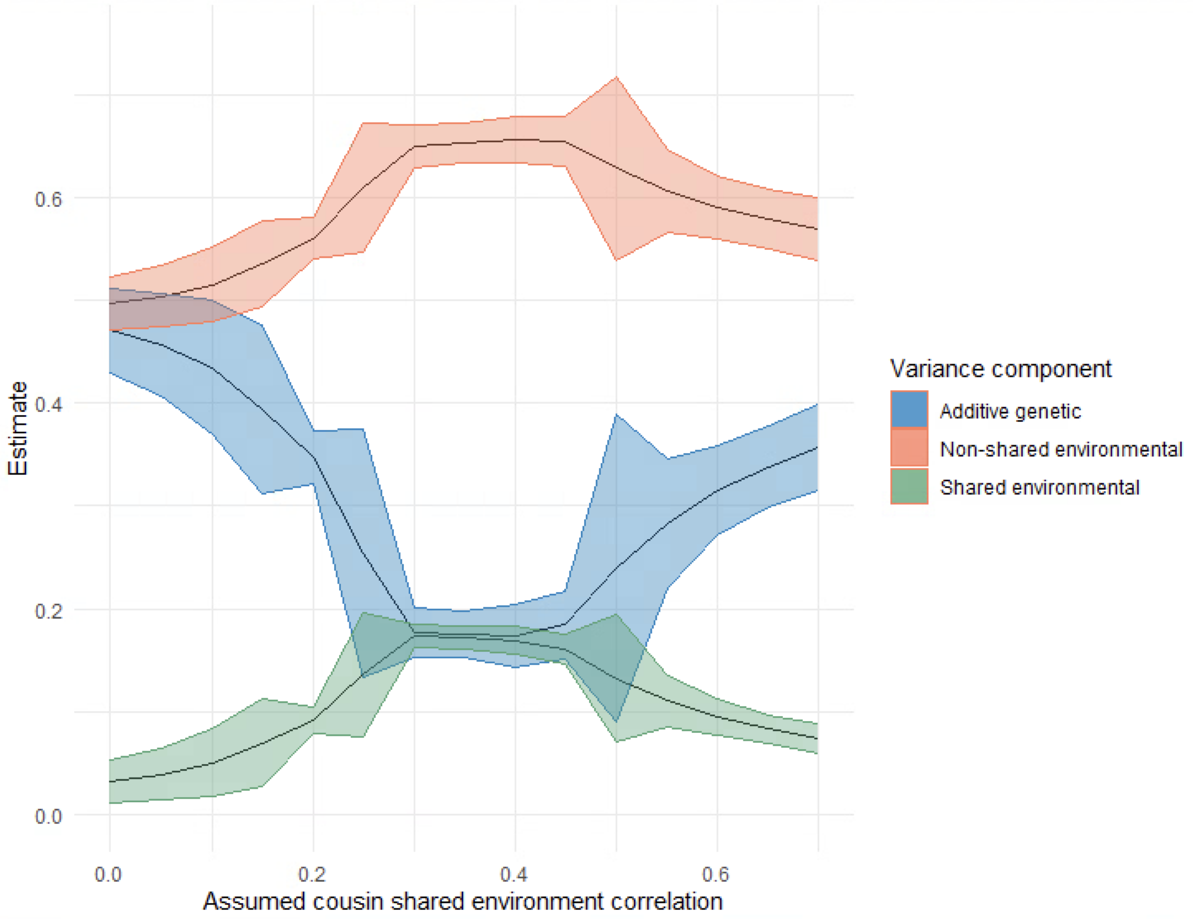

Figure S7: A, C, and E contribution to wealth under different shared environment correlation assumptions. Additive genetic (h^2), share environmental (c^2), and non-shared environmental (e^2) effects on wealth across assumptions of first cousin shared environment correlation. Sibling shared environment correlation was assumed to be 1. Intervals between assumptions are .05 and ribbons represent 95% confidence intervals.

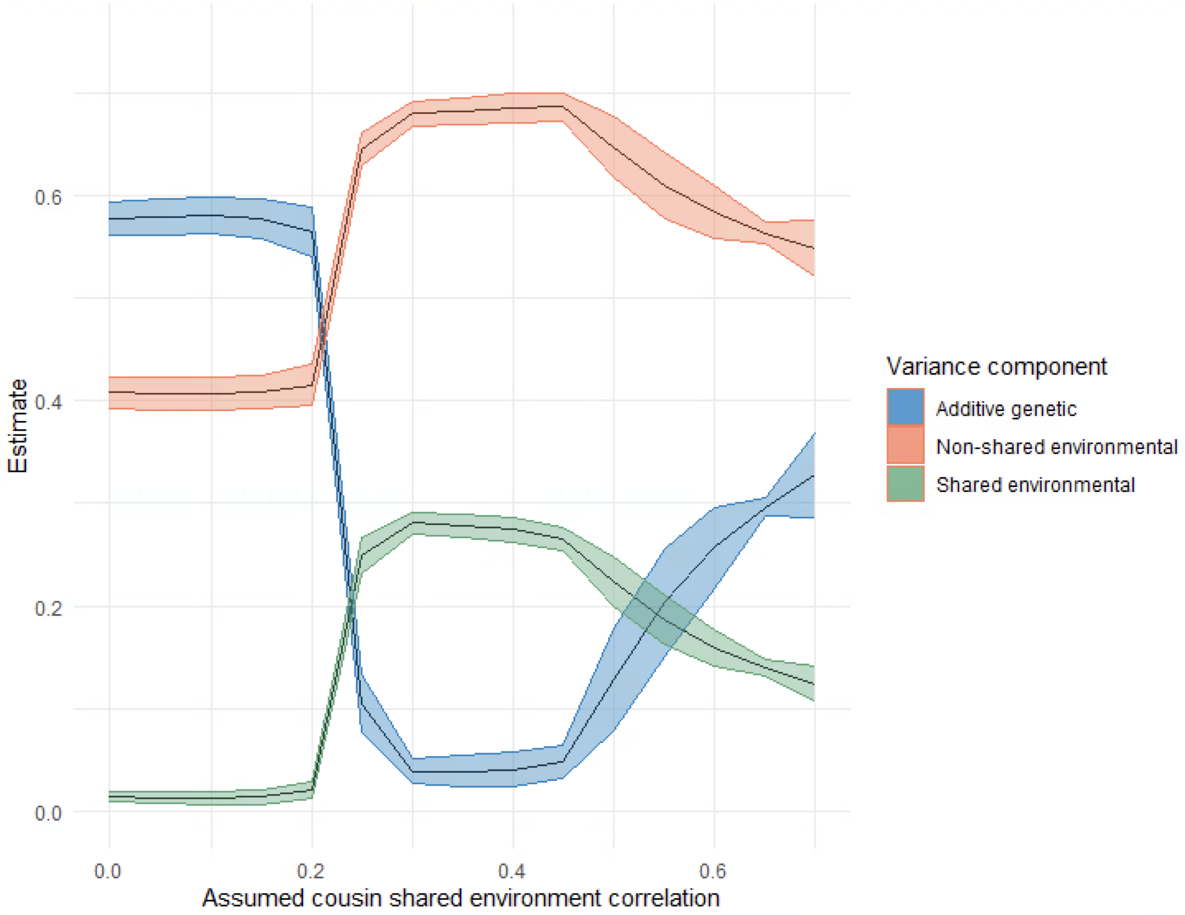

Correlations

Table S4: Phenotypic correlations between education, occupation, income, and wealth.

	Education	Occupation	Income	Wealth
Education	1	.58	.24	.27
Occupation		1	.38	.29
Income			1	.37
Wealth				1

Table S5: Family pedigree ACE (sibling r_c=1, estimated cousin r_c=.59) additive genetic correlations between education, occupation, income, and wealth.

	Education	Occupation	Income	Wealth
Education	1	.47	.35	.90
Occupation		1	.59	.58
Income			1	.39
Wealth				1

Table S6: Family pedigree ACE (sibling r_c =1, cousin r_c =0) additive genetic correlations between education, occupation, income, and wealth.

	Education	Occupation	Income	Wealth
Education	1	.54	.53	.93
Occupation		1	.64	.63
Income			1	.58
Wealth				1

Table S7: IBD AE additive genetic correlations between education, occupation, income, and wealth.

	Education	Occupation	Income	Wealth
Education	1	.59	.59	.93
Occupation		1	.73	.69
Income			1	.66
Wealth				1

Table S8: GREML genetic correlations between education, occupation, income, and wealth.

	Education	Occupation	Income	Wealth
Education	1	.96 (.02)	.71 (.06)	.77 (.04)
Occupation		1	.79 (.05)	.79 (.05)
Income			1	.92 (.07)
Wealth				1

Table S9: LD score regression genetic correlations between education, occupation, income, and wealth.

	Education	Occupation	Income	Wealth
Education	1	.95 (.04)	.81 (.04)	.83 (.04)
Occupation		1	.90 (.05)	.82 (.05)
Income			1	.83 (.06)
Wealth				1

Table S10: Results for both Family pedigree ACE models' shared environment correlations between education, occupation, income, and wealth.

	Education	Occupation	Income	Wealth
Education	1	1	1	1
Occupation		1	1	1
Income			1	1
Wealth				1

Table S11: Family pedigree ACE (sibling r_c =1, estimated cousin r_c =.59) non-shared environment correlations between education, occupation, income, and wealth.

	Education	Occupation	Income	Wealth
Education	1	.07	.05	.30
Occupation		1	.16	.28
Income			1	.12
Wealth				1

Table S12: Family pedigree ACE (sibling r_c =1, cousin r_c =0) non-shared environment correlations between education, occupation, income, and wealth.

	Education	Occupation	Income	Wealth
Education	1	0	-.09	.16
Occupation		1	.12	.23
Income			1	.01
Wealth				1

Table S13: IBD AE non-shared environment correlations between education, occupation, income, and wealth.

	Education	Occupation	Income	Wealth
Education	1	-.01	-.12	.08
Occupation		1	.11	.23
Income			1	-.02
Wealth				1

Table S14: GREML residual correlations between education, occupation, income, and wealth.

	Education	Occupation	Income	Wealth
Education	1	.46	.20	.19
Occupation		1	.40	.23
Income			1	.28
Wealth				1

Parallel analysis

Figure S8: Parallel analysis scree plot of phenotypic correlation matrix.

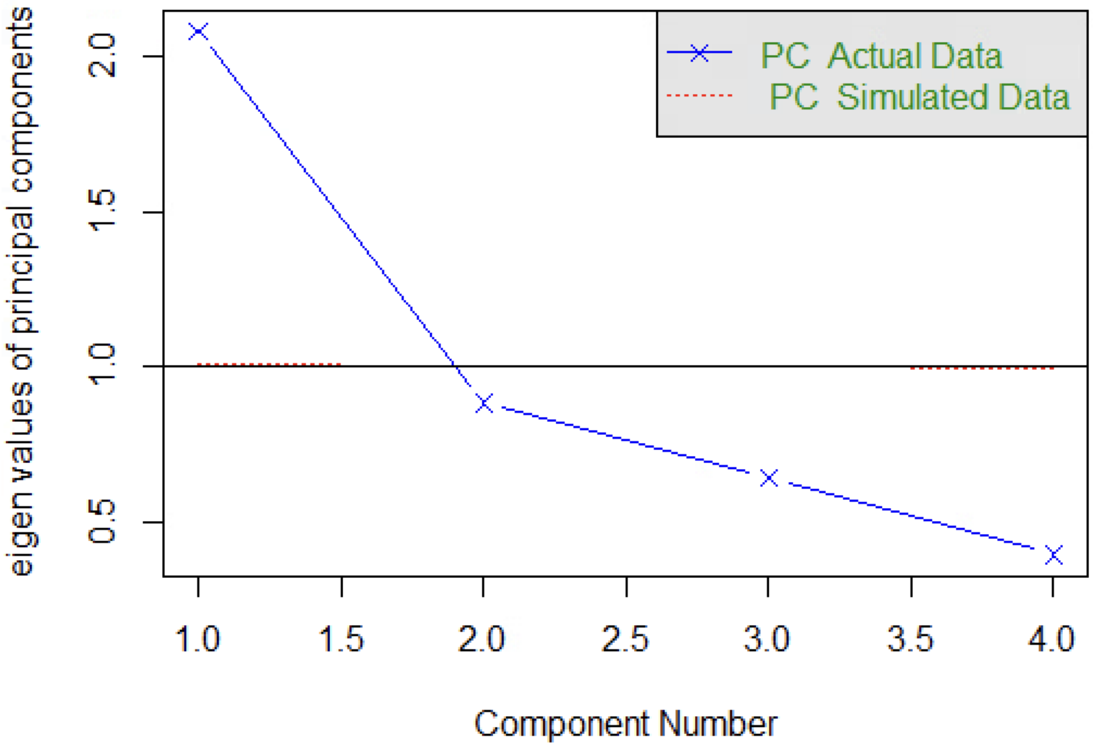

Figure S9: Parallel analysis scree plot of FP additive genetic correlation matrix with set rc=1 for full siblings and estimated rc=.59 cousins.

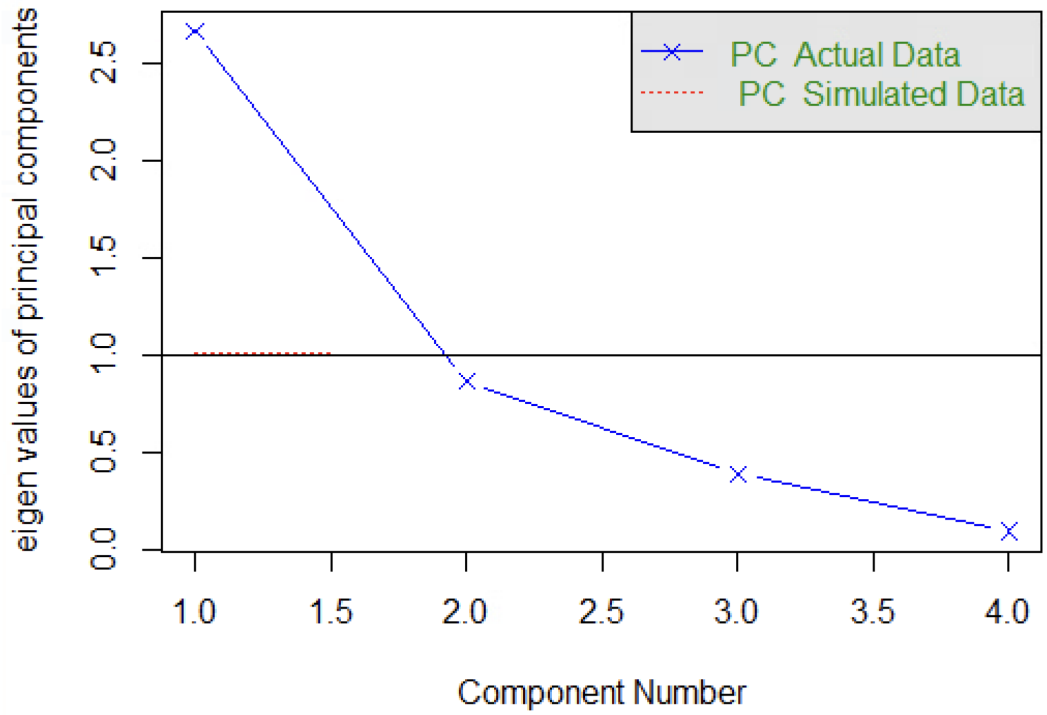

Figure S10: Parallel analysis scree plot of FP shared environment correlation matrix with set rc=1 for full siblings and estimated rc=.59 cousins.

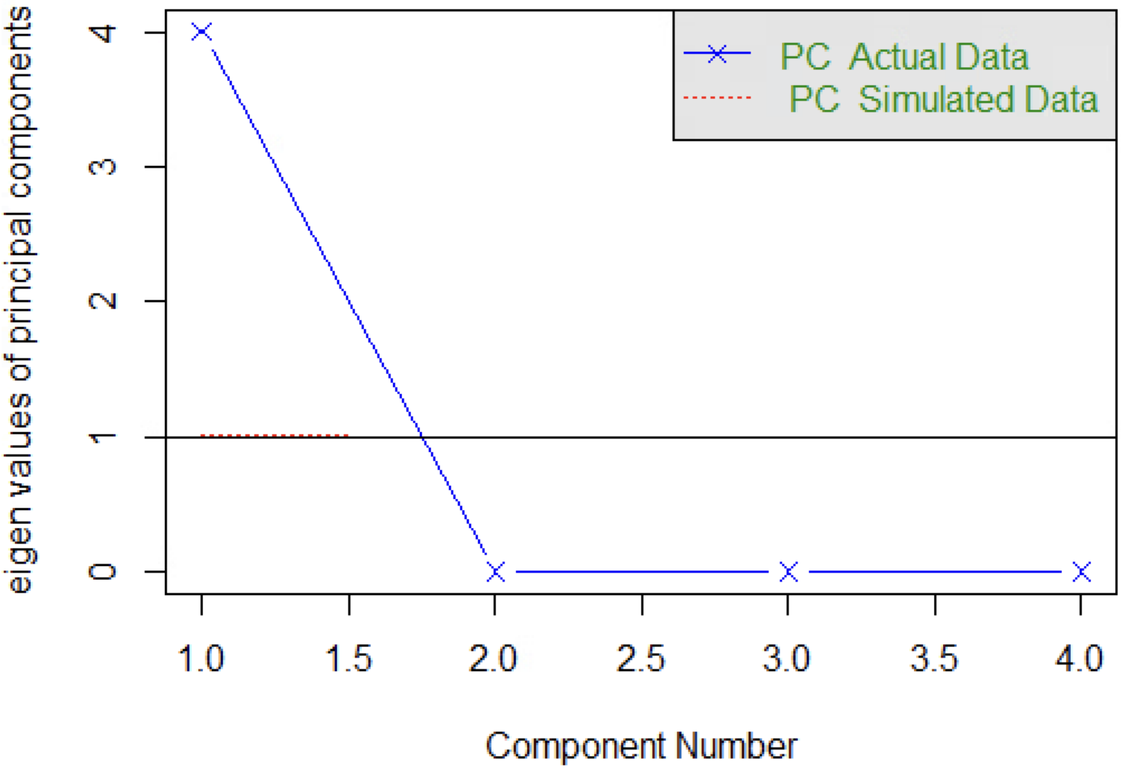

Figure S11: Parallel analysis scree plot of FP non-shared environment correlation matrix with set rc=1 for full siblings and estimated rc=.59 cousins.

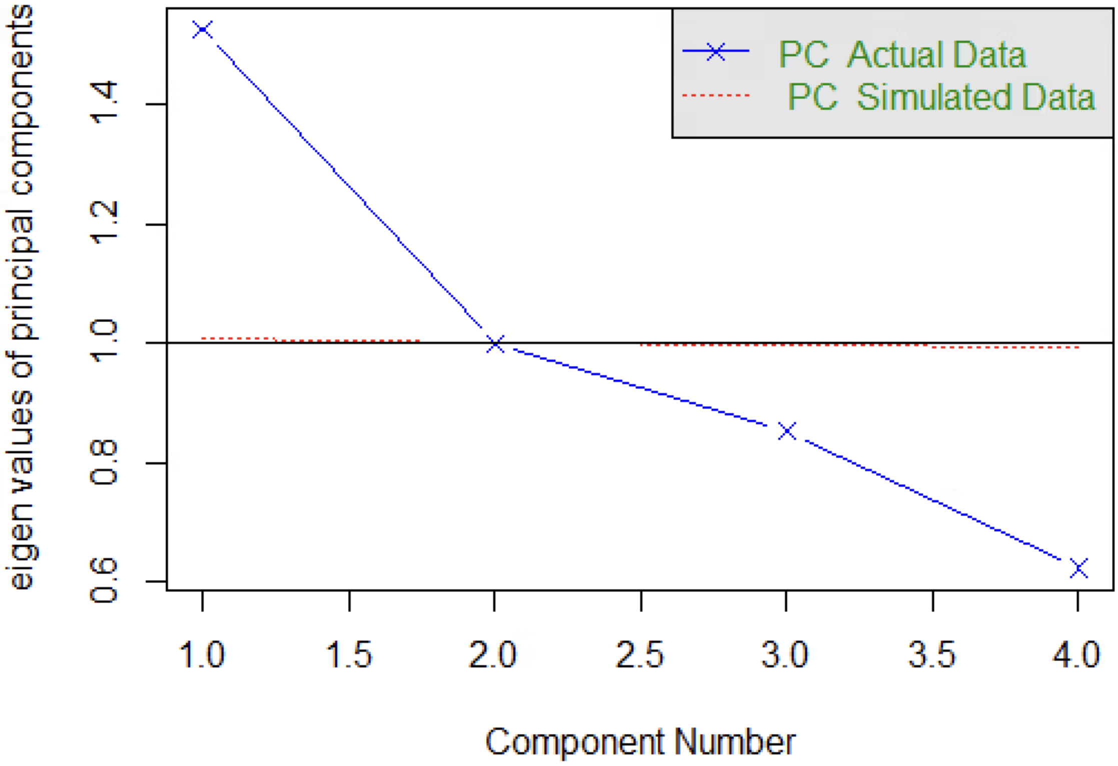

Figure S12: Parallel analysis scree plot of IBD additive genetic correlation matrix.

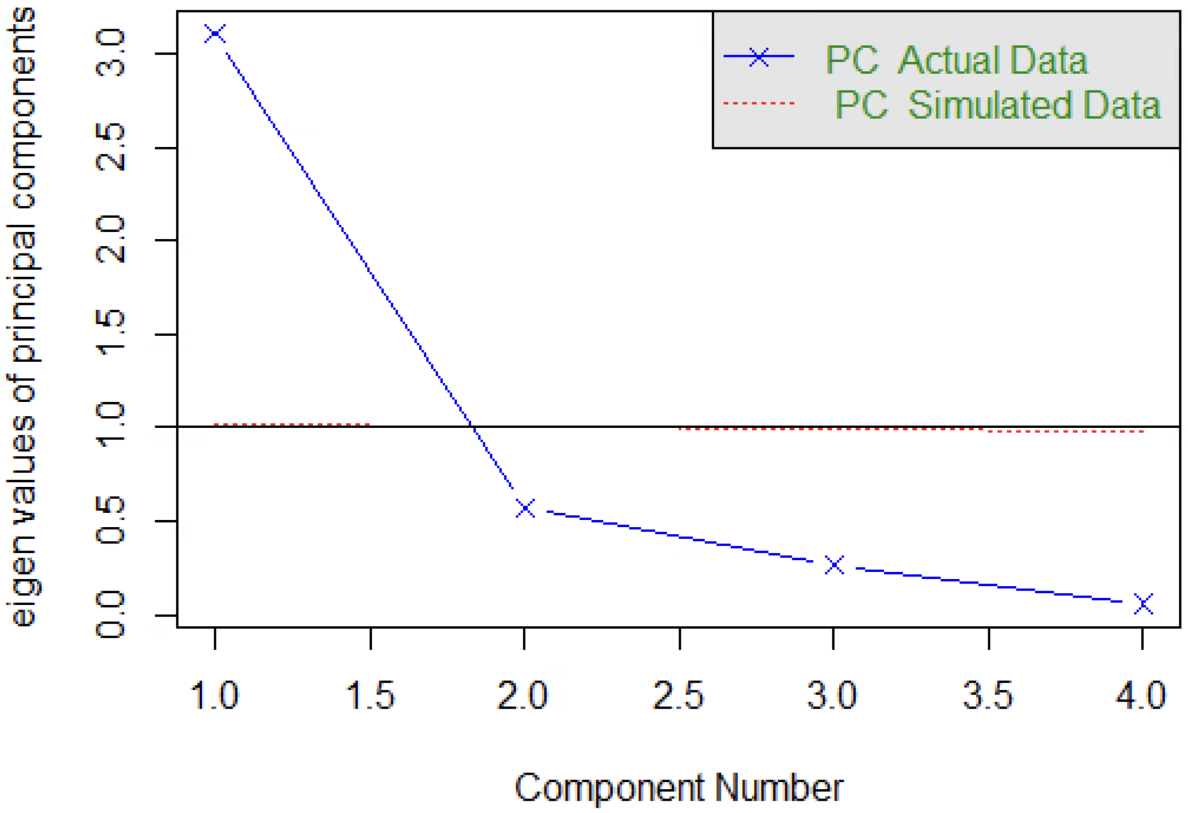

Figure S13: Parallel analysis scree plot of IBD non-shared environment correlation matrix.

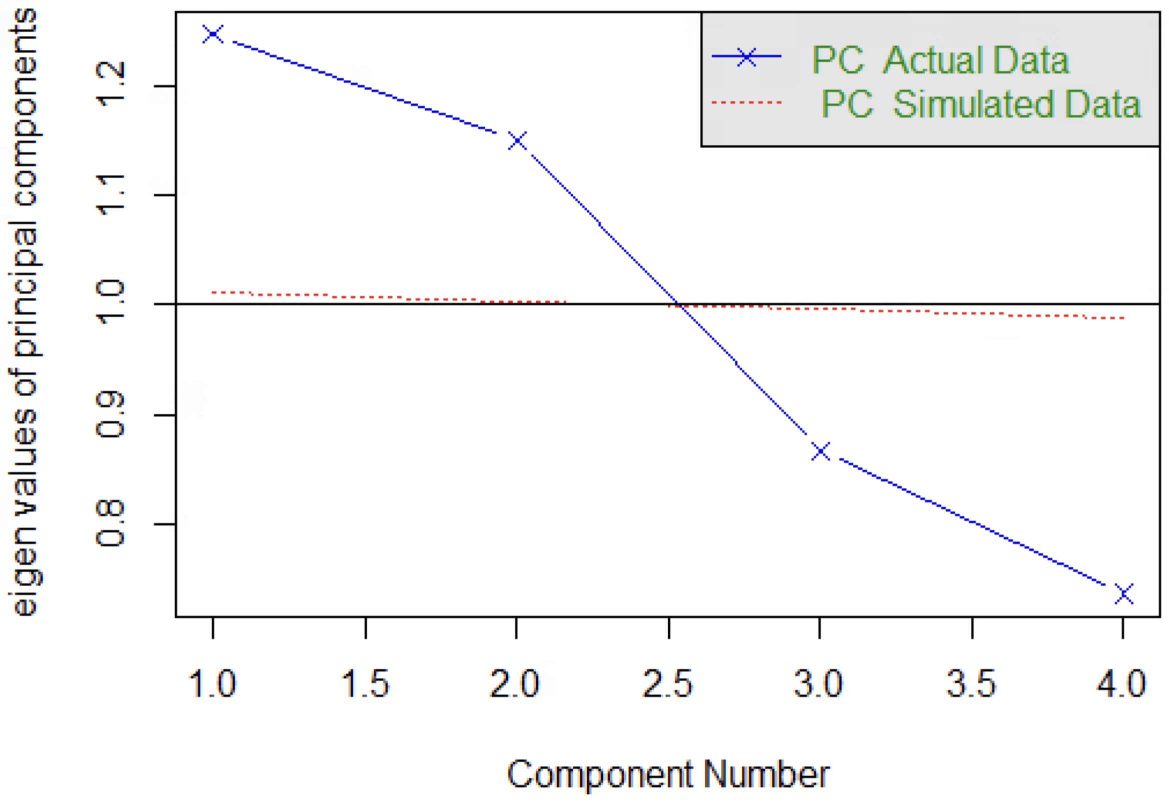

Figure S14: Parallel analysis scree plot of GREML additive genetic correlation matrix.

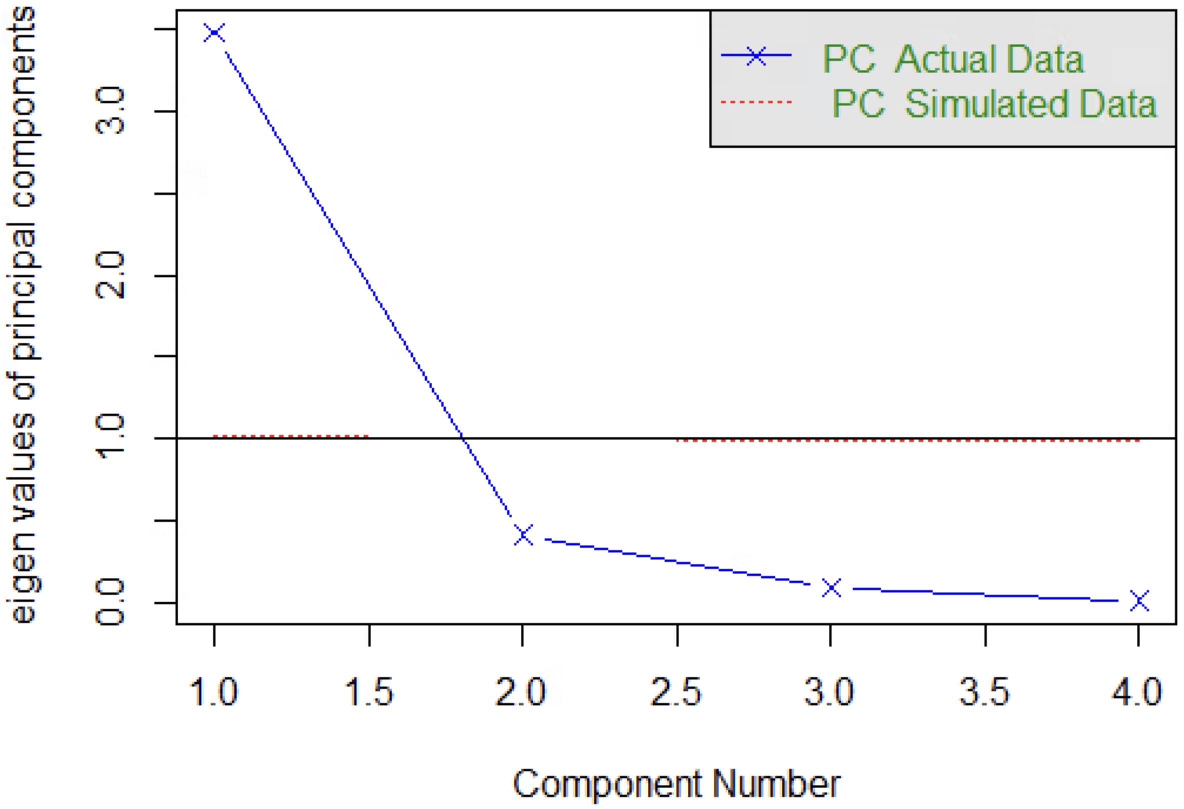

Figure S15: Parallel analysis scree plot of GREML residual correlation matrix.

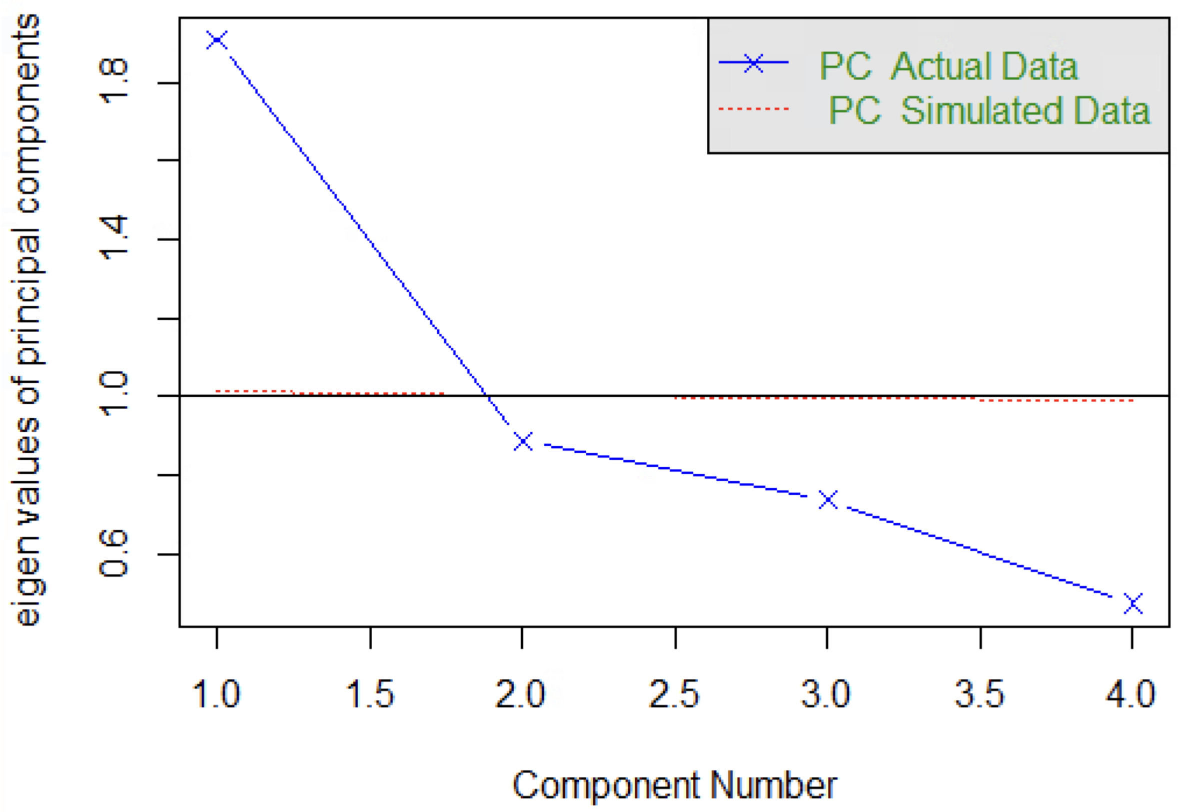

Figure S16: Parallel analysis scree plot of LD score regression correlation matrix.

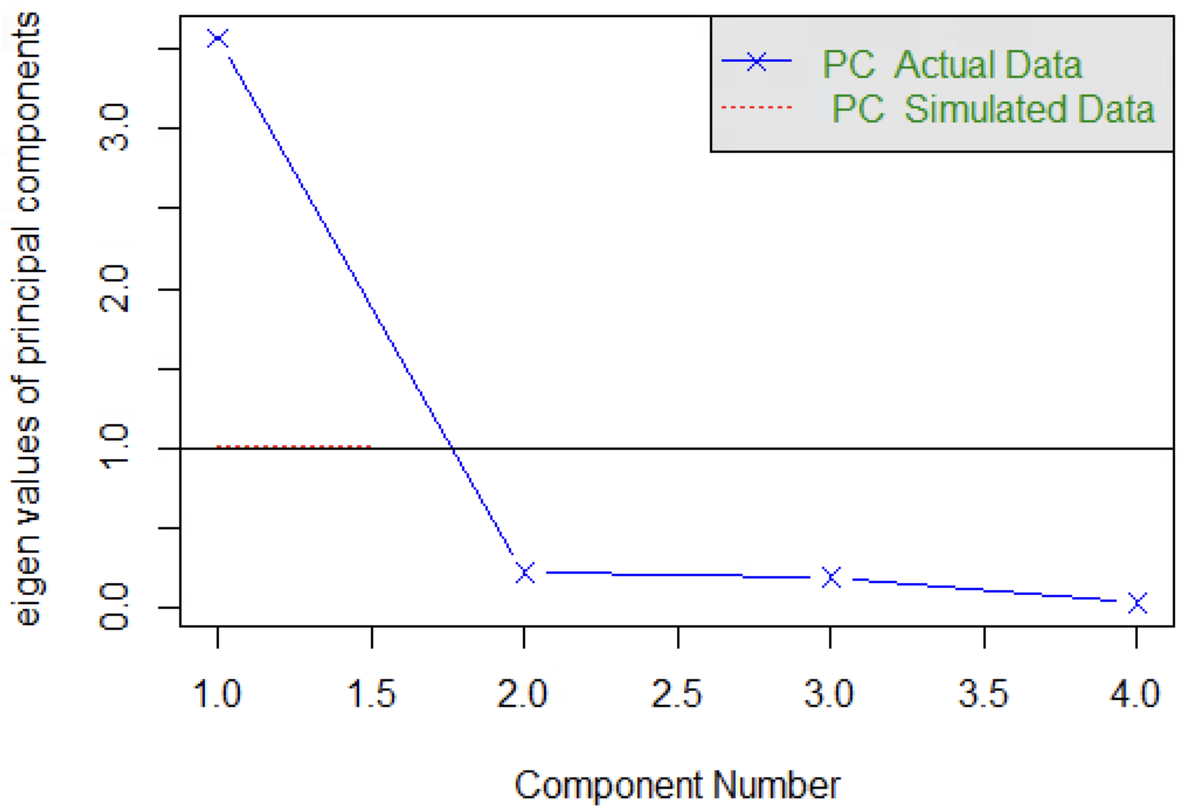

Explained variance and loadings of the principal components

Table S15: Principal component variance explained and loadings for additive genetic variance components (A).

Variance component	Additive genetic	Additive genetic	Additive genetic	Additive genetic
Method	FP ACE (Sib rc=1, Cousin rc=.59)	IBD AE	GREML	LD score regression
PC	1st	1st	1st	1st
Variance explained	66.5%	77.5%	86.8%	89.1%
Loadings				
Education	.86	.89	.92	.95
Occupation	.80	.85	.95	.97
Income	.68	.84	.92	.94
Wealth	.91	.94	.93	.92

Table S16: Principal component variance explained and loadings for non-shared environmental variance components (E). The loadings of the principal components in the non-shared environment differed in the FP model as a function of the modelling of the shared environment, making the non-shared environments of cousins and siblings difficult to capture.

Variance component	E	E	E
Method	FP ACE (Sib rc=1, Cousin rc=.59)	IBD AE	IBD AE
PC	1st	1st	2nd
Variance explained	38.1%	31.1%	28.7%
Loadings			
Education	.58	.03	.73
Occupation	.63	.79	-.08
Income	.44	.30	-.69
Wealth	.77	.71	.34

Table S17: Principal component variance explained and loadings for phenotypic, shared environmental and GREML residual variance components.

Variance component	Phenotypic	Shared environment	Residual
Method		FP ACE	GREML
PC	1st	1st	1st
Variance explained	52.0%	100%	47.6%
Loadings			
Education	.75	1.00	.68
Occupation	.81	1.00	.80
Income	.68	1.00	.69
Wealth	.64	1.00	.56

The systematicity of individual-specific environments

Part of the strengths of the family-based methods are that they can estimate correlations between environmental effects not shared within a family. An additional advantage with the applied IBD design, is the estimation of individual specific environments of full siblings while taking into account their empirical genetic similarity, and who arguably share more environmental influences than cousins. The retained non-shared environment principal components have split loadings on the 'big four', revealing individual specific structures with distinct features. Although the unique environment variance components include 'random' events, the principal component analysis indicated systematicity as indicated by the loadings of the principal components. The environments not shared between full siblings of the IBD method shows a first dimension with loading high on occupation and wealth (x-axis in Fig. S18), and the second low on income and high on education (y-axis in Fig. S18). The first combined wealth-occupation dimension and second opposite education-income dimension come from the family-based methods’ benefit of separating out non-shared variance. For the phenotypic, genetic and shared environmental principal components, the loadings were equal on the 'big four' indices (supplementary information), yielding a joined, general socioeconomic dimension. These principal components that explain much, if not all, of the variance (52-100%) and their equal loadings to all four indices resemble theoretical unidimensional models of SES.

Figure S17: Rotation of nonshared environmental principal components. Principal components of the non-shared environment of full siblings in the IBD model. The first dimension on the x axis and second dimension on the y axis with variance explained in parenthesis.

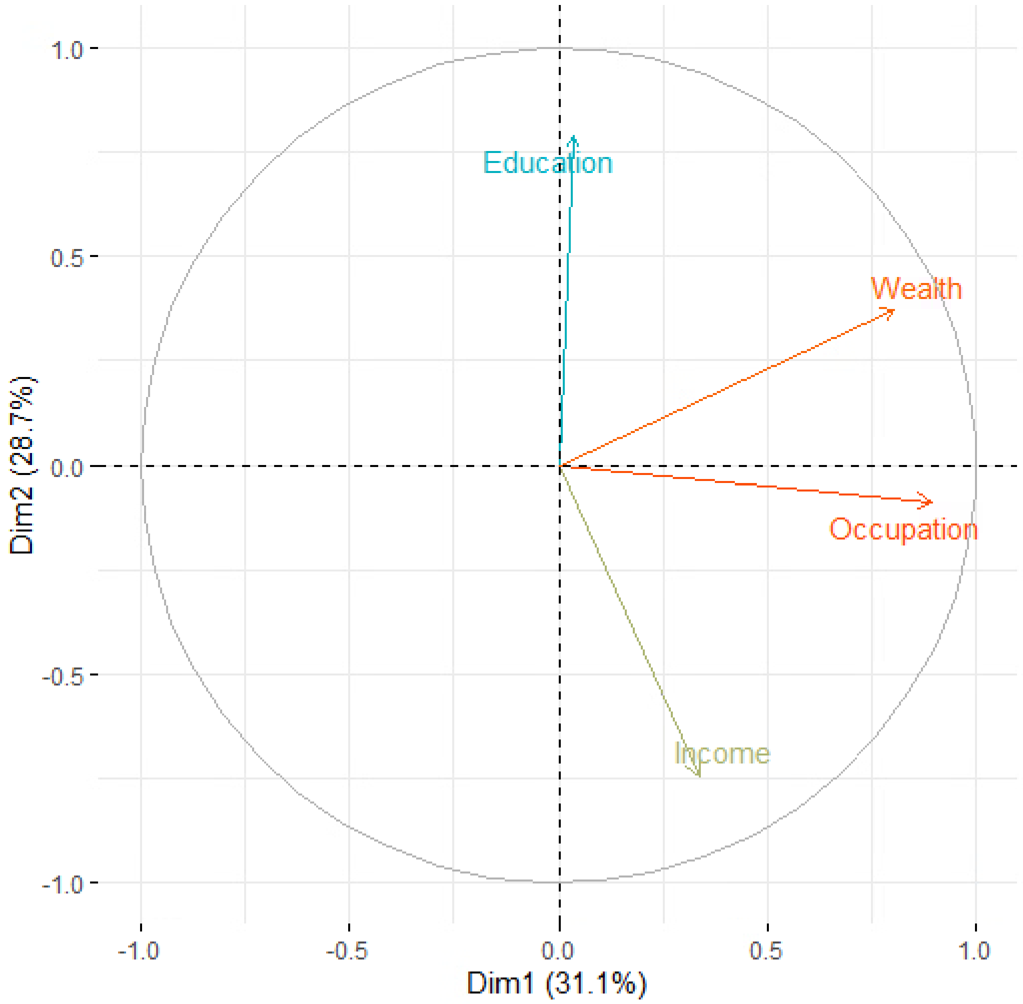

Supplement: Supplementary file 1 — Supplementary Information [file 41467_2025_58961_MOESM1_ESM.pdf]
