# Supplementary material for: The genetic and environmental composition of socioeconomic status in Norway
Source: Nat Commun. 2025 May 14;16:4461. doi: 10.1038/s41467-025-58961-6 (PMC12078464; doi:10.1038/s41467-025-58961-6)
Supplement: Supplementary file 2 — Description of Additional Supplementary Files [file 41467_2025_58961_MOESM2_ESM.pdf]

Description of Additional Supplementary Files

Supplementary Data S1. Descriptives of the population, the MoBa sample, and the subsamples used in the four methods
