# Supplementary material for: The genetic and environmental composition of socioeconomic status in Norway
Source: Nat Commun. 2025 May 14;16:4461. doi: 10.1038/s41467-025-58961-6 (PMC12078464; doi:10.1038/s41467-025-58961-6)
Supplement: Supplementary file 4 — Reporting Summary [file 41467_2025_58961_MOESM4_ESM.pdf]

nature portfolio

Corresponding author(s): Joakim C. Ebeltoft

Last updated by author(s): 19/03/25

Reporting Summary

Nature Portfolio wishes to improve the reproducibility of the work that we publish. This form provides structure for consistency and transparency in reporting. For further information on Nature Portfolio policies, see our Editorial Policies and the Editorial Policy Checklist.

Statistics

For all statistical analyses, confirm that the following items are present in the figure legend, table legend, main text, or Methods section.

n/a	Confirmed	
☐	☒	The exact sample size (n) for each experimental group/condition, given as a discrete number and unit of measurement
☐	☒	A statement on whether measurements were taken from distinct samples or whether the same sample was measured repeatedly
☐	☒	The statistical test(s) used AND whether they are one- or two-sided<br>*Only common tests should be described solely by name; describe more complex techniques in the Methods section.*
☐	☒	A description of all covariates tested
☐	☒	A description of any assumptions or corrections, such as tests of normality and adjustment for multiple comparisons
☐	☒	A full description of the statistical parameters including central tendency (e.g. means) or other basic estimates (e.g. regression coefficient) AND variation (e.g. standard deviation) or associated estimates of uncertainty (e.g. confidence intervals)
☒	☐	For null hypothesis testing, the test statistic (e.g. F, t, r) with confidence intervals, effect sizes, degrees of freedom and P value noted<br>*Give P values as exact values whenever suitable.*
☒	☐	For Bayesian analysis, information on the choice of priors and Markov chain Monte Carlo settings
☒	☐	For hierarchical and complex designs, identification of the appropriate level for tests and full reporting of outcomes
☒	☐	Estimates of effect sizes (e.g. Cohen's d, Pearson's r), indicating how they were calculated

Our web collection on statistics for biologists contains articles on many of the points above.

Software and code

Policy information about availability of computer code

Data collection	No software was used for data collection.
Data analysis	OpenMx package in R was used for heritability and multivariate covariance analysis. GCTA software was applied to create GRMs, perform GREML heritability and bivariate analyses. GCTA software was used to perform fastGWA. GWA SNP heritability was estimated using LD score regression software and GWA genetic correlations were performed in GenomicSEM R package.<br><br>The code used in this study is available at: https://github.com/joakimce/composition_ses.

For manuscripts utilizing custom algorithms or software that are central to the research but not yet described in published literature, software must be made available to editors and reviewers. We strongly encourage code deposition in a community repository (e.g. GitHub). See the Nature Portfolio guidelines for submitting code & software for further information.

Data

Policy information about availability of data

All manuscripts must include a data availability statement. This statement should provide the following information, where applicable:

- Accession codes, unique identifiers, or web links for publicly available datasets
- A description of any restrictions on data availability
- For clinical datasets or third party data, please ensure that the statement adheres to our policy

The data applied in the study are not publicly available. Instructions for access to MoBa data from the Norwegian Institute of Public Health can be found here: https://www.fhi.no/en/studies/moba/for-forskere-artikler/research-and-data-access/. Instructions for access to Statistics Norway data can be found here: https://www.ssb.no/en/data-til-forskning.

Research involving human participants, their data, or biological material

Policy information about studies with human participants or human data. See also policy information about sex, gender (identity/presentation), and sexual orientation and race, ethnicity and racism.

Reporting on sex and gender	We primarily use the term 'sex' because the data were obtained from Norwegian Medical Birth Register e.g., "... covariates consisting of 24 principal components, batch, age, and sex."
Reporting on race, ethnicity, or other socially relevant groupings	We primarily use the term 'ancestry', e.g. , "To identify a sub-population of European-associated ancestry, principal component analysis (PCA) was performed with 1000 Genomes phase 1 after LD-pruning."
Population characteristics	We use population-wide register data on adults age 35-45 who had children born between 1999 and 2008 from Norway.
Recruitment	Pregnant women were recruited from across Norway from 1999 to 2009 for the Norwegian Mother, Father and Child Cohort Study (MoBa).
Ethics oversight	The establishment of MoBa and initial data collection was based on a licence from the Norwegian Data Protection Agency and approval from The Regional Committees for Medical and Health Research Ethics. The MoBa cohort is now based on regulations related to the Norwegian Health Registry Act. The current study was approved by The Regional Committees for Medical and Health Research Ethics (project # 2017/2205).

Note that full information on the approval of the study protocol must also be provided in the manuscript.

Field-specific reporting

Please select the one below that is the best fit for your research. If you are not sure, read the appropriate sections before making your selection.

☐ Life sciences ☒ Behavioural & social sciences ☐ Ecological, evolutionary & environmental sciences

For a reference copy of the document with all sections, see nature.com/documents/nr-reporting-summary-flat.pdf

Behavioural & social sciences study design

All studies must disclose on these points even when the disclosure is negative.

Study description	Quantitative research
Research sample	The sample is a population-wide sample of parents from the Norwegian Mother, Father and Child Cohort Study (MoBa), recruited and managed by the Norwegian Institute of Public Health. Sample was linked with registry data restricted to age 35-45, an age in which their socioeconomic position is formative for the next generation. The Moba sample has slightly higher socioeconomic status compared to the Norwegian population (see supplementary information). This limits the generalizability to both the population and other demographic groups.
Sampling strategy	Our sampling strategy was to maximize the number of participants within the MoBa cohort to enable comparison of methods within the same sample and have sufficient sample size to power genomic designs.
Data collection	We used previously collected data, with collection and recruitment described elsewhere. Phenotypes used were recorded through linked administrative data. Education data: http://www.ssb.no/en/utdanning/norwegian-standard-classification-of-education/. Occupation data: https://www.ssb.no/en/arbeid-og-lonn/sysselsetting/statistikk/sysselsetting-registerbasert. Incoma and wealth data: https://www.ssb.no/en/inntekt-og-forbruk/inntekt-og-formue/statistikk/inntekts-og-formuesstatistikk-for-husholdninger. Data collection was prior to initiation of this study. The current analysts were not blinded to study hypotheses.
Timing	Education, occupation, income, and wealth register data includes the entire population of Norway, year-by-year. We used the most recently updated data available from 2022 (occupation) and 2023 (education, income, and wealth) on adults aged 35-45.

Data exclusions	We excluded participants on the grounds of missingness for phenotypic and genomic variables of study. We excluded participants with non-european ancestry from GREML and GWA analyses.
Non-participation	Not applicable
Randomization	Not applicable, participants were not allocated into experimental groups.

Reporting for specific materials, systems and methods

We require information from authors about some types of materials, experimental systems and methods used in many studies. Here, indicate whether each material, system or method listed is relevant to your study. If you are not sure if a list item applies to your research, read the appropriate section before selecting a response.

Materials & experimental systems

n/a	Involved in the study
☒	☐ Antibodies
☒	☐ Eukaryotic cell lines
☒	☐ Palaeontology and archaeology
☒	☐ Animals and other organisms
☒	☐ Clinical data
☒	☐ Dual use research of concern
☒	☐ Plants

Methods

n/a	Involved in the study
☒	☐ ChIP-seq
☒	☐ Flow cytometry
☒	☐ MRI-based neuroimaging

Plants

Seed stocks	*Report on the source of all seed stocks or other plant material used. If applicable, state the seed stock centre and catalogue number. If plant specimens were collected from the field, describe the collection location, date and sampling procedures.*
Novel plant genotypes	*Describe the methods by which all novel plant genotypes were produced. This includes those generated by transgenic approaches, gene editing, chemical/radiation-based mutagenesis and hybridization. For transgenic lines, describe the transformation method, the number of independent lines analyzed and the generation upon which experiments were performed. For gene-edited lines, describe the editor used, the endogenous sequence targeted for editing, the targeting guide RNA sequence (if applicable) and how the editor was applied.*
Authentication	*Describe any authentication procedures for each seed stock used or novel genotype generated. Describe any experiments used to assess the effect of a mutation and, where applicable, how potential secondary effects (e.g. second site T-DNA insertions, mosiacism, off-target gene editing) were examined.*
